# Sevoflurane Post-conditioning Enhanced Hippocampal Neuron Resistance to Global Cerebral Ischemia Induced by Cardiac Arrest in Rats through PI3K/Akt Survival Pathway

**DOI:** 10.3389/fncel.2016.00271

**Published:** 2016-11-30

**Authors:** Zhihua Wang, Zhi Ye, Guoqing Huang, Na Wang, E. Wang, Qulian Guo

**Affiliations:** ^1^Department of Anesthesiology, Affiliated Xiangya Hospital of Central South UniversityChangsha, China; ^2^Department of Anesthesiology, Hainan General HospitalHaikou, China; ^3^Emergency Department, Affiliated Xiangya Hospital of Central South UniversityChangsha, China

**Keywords:** sevoflurane, post-conditioning, PI3K/Akt, global cerebral ischemic injury, mitochondrial biogenesis

## Abstract

The purpose of this current study was to evaluate whether improvement of mitochondrial dysfunction was involved in the therapeutic effect of sevoflurane post-conditioning in global cerebral ischemia after cardiac arrest (CA) via the PI3K/Akt pathway. In the first experiment, animals were randomly divided into three groups: a sham group, a CA group, a CA+sevoflurane post-conditioning group (CA+SE). Sevoflurane post-conditioning was achieved by administration of 2.5% sevoflurane for 30 min after resuscitation. Sevoflurane post-conditioning has a significant neuroprotective effect by increasing survival rates and reducing neuronal apoptosis. Additionally, the gene and protein expression of PGC-1α, NRF-1, and TFAM, the master regulators of mitochondrial biogenesis, were up-regulated in the CA+SE group, when compared to the CA group. Similarly, in contrast to the CA group, mitochondria-specific antioxidant enzymes, including heat-shock protein 60 (HSP60), peroxiredoxin 3 (Prx3), and thioredoxin 2 (Trx2) were also increased in the CA+SE group. Finally, administration of sevoflurane ameliorated mitochondrial reactive oxygen species (ROS) formation and maintained mitochondrial integrity. In the second experiment, we investigated the relationship between the PI3K/Akt pathway and mitochondrial biogenesis and mitochondria-specific antioxidant enzymes in sevoflurane-induced neuroprotection. The selective PI3K inhibitor wortmannin not only eliminated the beneficial biochemical processes of sevoflurane by reducing the level of mitochondrial biogenesis-related proteins and aggravating mitochondrial integrity, but also reversed the elevation of mitochondria-specific antioxidant enzymes induced by sevoflurane. Therefore, our data suggested that sevoflurane post-conditioning provides neuroprotection via improving mitochondrial biogenesis and integrity, as well as increasing mitochondria-specific antioxidant enzymes by a mechanism involving the PI3K/Akt pathway.

## Introduction

A brief period of global brain ischemia, such as that induced by cardiac arrest (CA) or cardiopulmonary bypass surgery can occur under various perioperative circumstances and result in long-term neurological disability or death. Compelling evidences have shown that reperfusion after global cerebral ischemia causes severe damage to mitochondria (Zhou et al., 2011; Jiang et al., 2014; Park et al., 2015). It's well-known that mitochondria play an essential and pivotal role in cellular bioenergetics by regulating energy metabolism, generating reactive oxygen species (ROS), and mediating apoptosis in response to cerebral ischemia (Guo et al., 2016). Previous studies have suggested that mitochondrial dysfunction aggravates neuronal injury after ischemic reperfusion because nerve cells are greatly dependent on mitochondria to support their high energy demand (Rau et al., 2012; Miao et al., 2016) Thus, the causes of mitochondrial dysfunction in ischemic reperfusion are the objects of intense investigations, in view of possible therapeutic applications.

Several lines of evidence have pointed to altered mitochondrial biogenesis as one of the causal mechanisms of mitochondrial dysfunction, which occurred during the reperfusion period following cerebral ischemia in animals (Wang et al., 2014; Xie et al., 2014). Mitochondrial biogenesis is regulated by a network of signaling factors, including three most important ones as follows. Firstly, peroxisome proliferator-activated receptor γ coactivator-1α (PGC-1α) is a major regulator involved in mitochondrial biogenesis and plays an important role in oxidative metabolism in the brain (Kleiner et al., 2009). Secondly, nuclear respiratory factor 1 (NRF-1), the first isolated mammalian transcription factor common to the expression of nuclear respiratory genes, functions as a positive regulator of transcription (Scarpulla, 2002). Lastly, mitochondrial transcriptional factor A (TFAM) is involved in mitochondrial DNA (mtDNA) maintenance and drives the transcription and replication of mtDNA (Escrivá et al., 1999; Scarpulla, 2002).

Sevoflurane, a novel volatile anesthetic with minimal pungency, low solubility and less toxicity, is used widely in anesthetic practice. Recently, we have demonstrated that sevoflurane preconditioning and post-conditioning could alleviate ischemic reperfusion injury in the brain (Ye et al., 2012a,b,c, 2015). However, the mechanism of sevoflurane's protective effect on cerebral ischemia in the context of mitochondrial dysfunction has not been well-investigated. Additionally, recent studies demonstrated that some specific antioxidant enzymes are located in mitochondria, such as heat shock protein 60 (HSP60), peroxiredoxin 3 (Prx3), and thioredoxin 2 (Trx2), which protect mitochondria against ROS-induced damage by catalyzing the reduction of H_2_O_2_ into water (Hwang et al., 2010; Lee et al., 2015). So far, it has not been well-established in a global cerebral ischemic model whether these mitochondria-specific antioxidant enzymes in the hippocampus mediates the neuroprotective effect of sevoflurane.

Mitochondrial dysfunction was characterized by loss of mitochondrial membrane potential (MMP) and opening of mitochondrial permeability transition pore (MPTP), and initiated mitochondria-mediated apoptosis, a pathological response to hypoxia-ischemia injury (Gong et al., 2013). Hence, it will be interesting to further explore whether sevoflurane post-conditioning plays a role in maintaining mitochondrial structural integrity after global cerebral ischemia.

Previous studies have indicated that phosphatidylinositol-3-kinase (PI3K)/Akt pathway, an anti-apoptotic pro-survival kinase signaling cascade, plays a pivotal role in anesthetic post-conditioning. Recently, we have demonstrated that the neuroprotective effect from sevoflurane post-conditioning may be due to the activation of the PI3K/Akt survival pathway and the inhibition of neuronal apoptosis (Ye et al., 2015). Whether the PI3K/Akt pathway is also essential for mitochondrial biogenesis-mediated sevoflurane post-conditioning has yet to be investigated.

In the present study, therefore, we used a CA-induced global cerebral ischemic *in vivo* model to investigate whether sevoflurane post-conditioning is capable of promoting neuronal survival after global cerebral ischemia via preserving mitochondrial biogenesis and integrity and increasing expression of mitochondria-specific antioxidant enzymes, and whether this function is regulated by a mechanism involving the PI3K/Akt survival pathway.

## Materials and methods

### Animals

Adult male Sprague-Dawley (SD) rats, weighing 300–400 g, were used in the experiments. The rats were kept at room temperature with a 12 h light/dark cycle following surgery. They had access to food and water *ad libitum* and were food-deprived for 12 h before surgery. The study was approved by the ethics committee of Central South University and conducted according to the National Institutes of Health (NIH) Guide. All efforts were made to minimize animal suffering during the experiments.

### Establishment of a CA animal model

A rat model of CA was established by the delivery of alternating current between the esophagus and the chest wall to induce ventricular fibrillation based on our previous study (Huang et al., 2013). All animals were fasted but given free access to water on the night prior to the experiment. All animals were anesthetized by single dose intraperitoneal injection of chloral hydrate (350 mg/kg, IP). After trachea intubation, all animals were mechanically ventilated (Harvard Model 683 Small Animal Ventilator, Harvard Apparatus, Massachusetts) with a mixture of N_2_O and O_2_ (70:30%) at a tidal volume of 8 ml/kg, a breathing rate of ~60 min^−1^, adapted to maintain normocapnia (assessed by blood gas analyses) and no positive end-expiratory pressure. Polyethylene catheters were inserted into the left femoral artery and vein to monitor arterial blood pressure or inject drugs. An electrocardiogram was recorded by three subcutaneous needle electrodes, and a tympanic membrane temperature (Tty) probe was used to maintain Tty at 37.5 ± 0.2°C by using a lamp and heating pad. A BL-420s multichannel physiological signal recording system (Chengdu Taimeng Science and Technology Co., Ltd., Chengdu, China) was used to record the electrocardiogram, rectal temperature and arterial blood pressure. Ventricular fibrillation was initiated using alternating current (12 V, 50 Hz) via an esophageal electrode and ventilation was stopped. CA was identified using the following criteria: (1) the systolic arterial pressure after electrical stimulation gradually fell to below 25 mmHg; and (2) pulsations in the arterial pressure waveform disappeared. After reaching the criteria for CA, electrical stimulation was performed for 2 min followed by 5 min of observation without treatment. After 7 min of CA, cardiopulmonary resuscitation (CPR) was started: 60 breaths/min (100% oxygen), external manual chest compression at a rate of 200 min^−1^, duty cycle 50%, and compression depth of 25% of the anterior-posterior diameter of the chest. After 2 min of CPR without restoration of spontaneous circulation (ROSC), a defibrillation attempt was performed with one biphasic shock of 1 Joule (M-Series, Zoll Corporation, Germany). CPR was continued and adrenaline administered (epinephrine, 20 μg/kg), if ROSC could not be achieved 30 s after the first defibrillation attempt. Defibrillation procedures were repeated after 30 s each. In case of 6 min of unsuccessful CPR, resuscitation was stopped and the animal was declared dead. ROSC was defined as maintenance of an unassisted MAP beyond values of 50 mmHg for at least 10 consecutive minutes. No further defibrillation or cardioversions, vasopressors or antiarrhythmic drugs were allowed after ROSC was achieved once. Breathing rate was adjusted to reach normocapnia 20 min after ROSC and maintain it until sufficient spontaneous ventilation and extubation. Application of sodium bicarbonate (8.4%) was titrated according to the blood gas analyses aiming at a base excess of 0 to –5 mmol/L, 20 min after ROSC. Animals without ROSC following standard cardiopulmonary resuscitation (CPR) for 15 min were defined as resuscitation failures.

### Experimental design

The rats were randomly assigned into three groups for the first experiment: a sham group (no CA), a CA group, and a CA+sevoflurane post-conditioning group (CA+SE). The core temperature of the rats was continuously measured with a rectal temperature probe, which was maintained at 37 ± 0.5°C using an infrared thermolamp until awake or 4 h after ROSC. Arterial and venous catheterization, anesthesia and endotracheal intubation were performed in the sham group. An esophageal electrode was implanted in the sham group with a length of 10 cm from the incisor, and then electrical stimulation using the same parameters was performed for 90 s to induce generalized twitching but not CA. In the CA groups, ventricular fibrillation was induced for 7 min and then standard CPR was performed. Sevoflurane post-conditioning group (CA+SE): rats were subjected to ischemia induced by cardiac arrest plus sevoflurane post-conditioning. Sevoflurane, which was delivered to gas admixture (oxygen) at a concentration of 2.5% via acalibrated vaporizer (Sevorane Vapor 15.3, Abbott), was administered after 60 min stable phase via an endotracheal tube for 30 min and the inhalational oxygen and sevoflurane concentrations were maintained and monitored constantly by a gas analyzer (Datex-Oheda, Helsinki, Finland).

**Figure 1:**
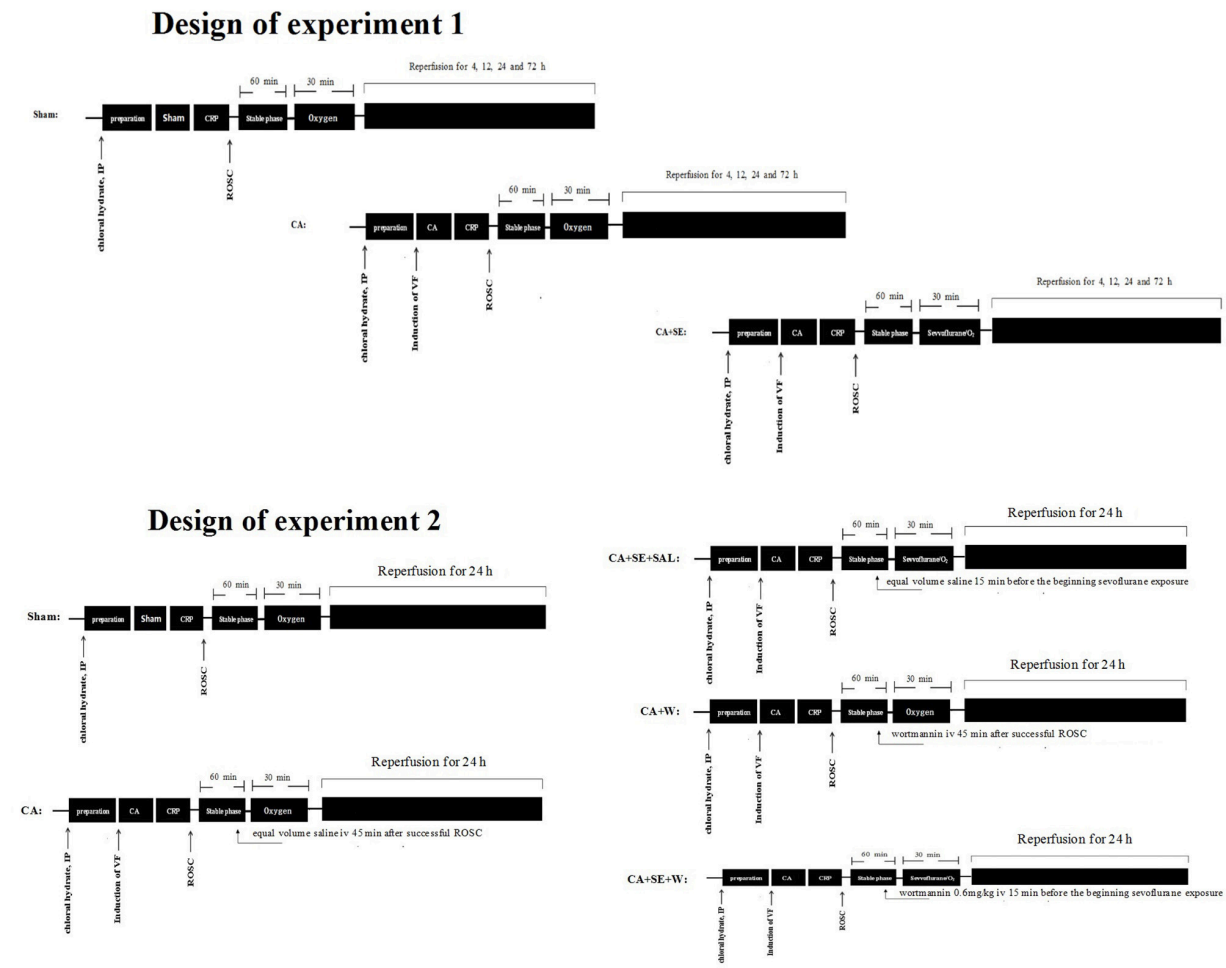
**Diagram of the experimental procedures**. IP, intraperitoneal injection; VF, ventricular fibrillation; CA, cardiac arrest; CRP, cardiopulmonary resuscitation; ROSC, restoration of spontaneous circulation; S, sham group; CA, cardiac arrest group; SE, Sevoflurane; W, Wortmannin.

In the second set of experiments, rats were randomly assigned into five separate experimental groups. In the sham (S) and CA groups, rats were subjected to the same procedures as those in the first part of this experiment. The rats in the wortmannin group (CA+W) were injected intravenously with wortmannin (Sigma, St. Louis, MO, USA) at 0.6 mg/kg 45 min after successful ROSC and the rest of the procedure was same as that described in the CA group. Wortmannin was dissolved in normal saline at a concentration of 5 ml/kg. In the sevoflurane post-conditioning group (CA+SE+SAL), equal volume saline was administered in the same manner 15 min before sevoflurane exposure and the rest of protocol was same as that described in CA+SE group. Sevoflurane plus wortmannin group (CA+SE+W) was administered intravenously with wortmannin 0.6 mg/kg 15 min before sevoflurane exposure via the right femoral vein and the rest of protocol was the same as that described in the SE group above (Figure 1).

### Nissl staining and TUNEL assay

Paraffin-embedded brain tissues were cut into 5 μm sections, and Nissl staining and TUNEL assays (Beyotime Institute of Biotechnology, Nantong, China) were performed for *in situ* detection of apoptosis. Five fields of vision (magnification of 400×) in the hippocampal CA1 area were randomly sampled from each brain tissue sample, and Nissl-stained neuronal cells were counted. For TUNEL assay, One Step TUNEL Apoptosis Assay Kit (Beyotime, China) was used according the manufacturer's instruction. For each section, the density of TUNEL-positive cells was expressed as the number of apoptotic cells per mm^2^. For each animal, the mean density of apoptotic cells was calculated in each region of the brain based on up to 5 sections.

### RNA isolation and cDNA synthesis and quantitative real-time PCR

Total RNA was isolated from frozen hippocampal samples using RNeasy Mini kit (Qiagen) according to the manufacturer's instructions. Five microgram RNA was used to synthesize the first strand of cDNA using random hexamer primers and SuperScript First-strand synthesis system for RT-PCR (Invitrogen). PCR was performed using SYBR Green PCR Master Mix (Applied Biosystems). Fluorescence was quantified using SDS v1.2x system software (Applied Biosystems). The forward and reverse primers used were: TFAM, GAAAGCACAAATCAAGAGGAG, CTGCTTTTCATCATGAGACAG; NRF-1, TTACTCTGCTGTGGCTGATGG, CCTCTGATGCTTGCGTCGTCT; PGC-1α, GTGCAGCCAAGACTCTGTATGG, GTCCAGGTCATTCACATCAAGTTC; and GADPH, GGGTCAGAAGGATTCCTATG, GGTCTCAAACATGATCTGGG.

### Mitochondrial DNA quantification

The mitochondrial DNA (mtDNA) copy number was measured using real-time PCR as described in a previous study (Edwards et al., 2010). The animals were decapitated at 4, 12, 24, and 72 h after reperfusion and the hippocampal tissue was isolated rapidly. Total DNA was extracted from the brain tissue using QIAamp DNA extraction kit (Qiagen, Shanghai, China). In total, 10 ng of genomic DNA was used for amplifying mtDNA and nuclear DNA markers. The mtDNA was amplified using primers specific for the mitochondrial cytochrome b (Cyt B) gene, and the mitochondrial DNA copy number was normalized to nuclear DNA copy number by amplifying β-actin nuclear gene. The oligonucleotides (probes) for TaqMan PCR were labeled with fluorescent reporter dye FAM (6-carboxyfluorescein) at the 5′ end and fluorescent BHQ-1 dye at the 3′ end. Real-time PCR amplification was performed using a LightCycler™ 480 II Probes Master kit (Roche Applied Science, Mannheim, Germany). The fluorescence threshold (Ct) value was calculated using LightCyclerTM 480 II Probes system software (Roche Applied Science, Germany), and the 2^−ΔΔCt^ method was used to calculate the relative levels of expression (Livak and Schmittgen, 2001). The primer and probe sequences are listed in Table 1.

**Table 1:** **Mitochondrial DNA sequences**.

**Gene**	**Primer sequence (5′–3′)**
CytB	Forward primer: TCCTGCATACTTCAAAACAACG
	Reverse primer: AACATICCGCCCAATCACCCAAA
	Probe: AACATTCCGCCCAATCACCCAAA
ß-actin	Forward primer: CTATGTTGCCCTAGACTTCGAGC
	Reverse primer: TTGCOGATAGTGATGACCTGAC
	Probe: CACTGCCGCATCCTCTTCCTCCC

### Isolation of mitochondrial fraction

The mitochondrial fraction of the harvested brain tissues (hippocampus) was prepared as previously described (Liao et al., 2014). The cytosolic and mitochondrial fractions of the harvested brain tissues were prepared. Each brain tissue sample was homogenized in ice-cold lysis buffer [200 mM mannitol, 80 mM HEPES-KOH (pH 7.4), and protease inhibitor cocktail] by 35 strokes of gentle pounding in a glass tissue grinder. Homogenates were centrifuged at 750 × g for 10 min at 4°C. After removing a portion of the supernatant, the remaining supernatant was centrifuged at 8000 × g for 20 min at 4°C. The mitochondrial fraction was obtained after washing the resultant pellets three times and resuspending the pellets in lysis buffer.

### Western blot

Hippocampal tissues were harvested from each of the two hemispheres at 24 and 72 h after reperfusion. Hippocampal protein extracts and Western blot analysis were performed as previously described (Kocki et al., 2015) using 20–50 μg of protein extract per lane. Equal amounts of protein were loaded in each well and subjected to 10% sodium dodecylsulfate-polyacrylamide gel electrophoresis (SDS-PAGE). Separated proteins were then transferred from the gel to polyvinylidene fluoride (PVDF; Millipore, Bedford, MA, USA) membranes and blocked in 5% non-fat dry milk prepared in 1× TBST. The membranes were incubated with primary antibodies overnight at 4°C. The membranes were incubated with primary antibodies for caspase-3 and -9; PGC-1α, NRF-1, and HSP 60 (all from Santa Cruz Biotechnology Inc.); TFAM, p-Akt (Ser^473^), Akt and GAPDH (Cell Signaling Technology, Beverly, MA, USA); Trx-2, Prx-3, and VDAC (Abcam, Cambridge, MA, USA). The membranes were washed with 1X TBST and the membranes incubated with appropriate secondary antibodies for 2 h at room temperature. The blots were developed using an ECL plus detection system (Beyotime Institute of Biotechnology) and relative band density was measured using FluorChem FC2 System (NatureGene Corp., USA).

### Measurement of mitochondrial ROS formation

ROS formation was assayed using a previously described dichlorodihydrofluorescein diacetate (DCFH-DA) assay (HaMai et al., 2001). In brief, 10 μl of mitochondria (5–10 μg) was suspended in 170 ml HEPES buffer (120 mM NaCl, 2.5 mM KCl, 1.2 mM NaH_2_PO_4_, 0.1 mM MgCl_2_, 5.0 mM NaHCO_3_, 6.0 mM glucose, 1.0 mM CaCl2, 10 mM HEPES, pH 7.4) in 96-well-plates; 20 ml of 100 mM DCFH-DA was added to each well, for a final volume of 200 ml. Fluorescence was read at an excitation wavelength of 485 nm and an emission wavelength of 530 nm using a fluorescent plate reader (Genios, TEC AN). Increased fluorescence intensity was considered to be indicative of an increase in intracellular ROS.

### Measurement of MMP

Changes in MMP were monitored in the presence of the fluorescent dye Rhodamine 123 (Rh123), as described previously (Emaus et al., 1986) with modifications. In brief, 10 μl of mitochondria (5–10 μg) was suspended in 90 μl of assay buffer (15 mM sucrose, 5 mM MgCl_2_, 5 mM sodium succinate, 5 mM K_2_HPO_4_, 20 mM HEPES, pH 7.4) in 96-well-plates and was incubated for 30 min in the presence of 20 μl of 100 μg/mL Rh123. The change of MMP was determined by measuring the fluorescence change in the reaction mixture at an excitation wavelength of 485 nm and an emission wavelength of 538 nm. The results were expressed as fluorescence intensities.

### Measurement of MPTP opening with calcein AM

MPTP opening was determined using a commercially available kit (GENMED SCIENTIFICS INC. U.S.A) according to the manufacturer's instructions. Isolated mitochondria were co-loaded with 1 mM calcein AM and 1 mM CoCl_2_ at room temperature in working solution (pH = 7.2). The working solution consists of 10 mM EGTA-CaEGTA buffer (free Ca^2+^ concentration 100 nM), 3 mM free Mg^2+^, 20 mM taurine, 0.5 mM dithiothreitol, 20 mM imidazole, 0.16 M potassium 4-morpholineethanesulfonate and 10 mg/ml fatty acid-free bovine serum albumin. MPTP opening was measured directly using a combination of calcein AM and CoCl_2_. Calcein AM, which fluoresces upon binding with Ca^2+^, was used to detect transient MPTP opening in isolated mitocchondria. This was achieved by monitoring changes in mitochondrial Ca^2+^ levels in the presence of CoCl_2_, which quenched the cytosolic Ca^2+^ signal produced by calcein AM. The fluorescence of calcein AM was monitored at an emission/excitation wavelength of 530/488 nm. The extent of Ca^2+^-induced MPTP opening was estimated by determining the difference in fluorescence intensity.

### Statistical analysis

All measurement data were expressed as mean ± standard deviation (*SD*), and all statistical analyses were performed using statistical software (SPSS version 13.0). Comparison of the same parameters among groups was done using one-way analysis of variance (ANOVA), and the difference between pairs of means was tested *post-hoc* with Fisher's Least Significant Difference (LSD) test. The difference in survival rates among the various groups was compared using Wilcoxon (Gehan) survival analysis. *P* < 0.05 was considered statistically significant.

## Results

### Physiological variability during the study

The physiological parameters (MAP, pH, and glucose) and levels of arterial blood gas tensions including PaCO_2_ and PO_2_ were monitored before, during, and after (1 and 3 h post-ROSC) CA (Table 2). The baseline parameters were similar across different animal groups with no significant difference. Additionally, there were no significant differences in body weight, basal body temperature, breathing frequency, and heart rate of the rats among three groups.

**Table 2:** **Physiological parameters**.

**Groups**	**Time**	**MAP (mmHg)**	**pH**	**PaCO_2_ (mmHg)**	**PaO_2_ (mmHg)**	**HR [bpm]**	**Glc (mg/dl)**
Sham	Baseline	110±6	7.41±0.03	41.5±4.8	141.7±14.8	272±13	167±22
	VF	105±3	7.43±0.02	40.7±2.9	154.7±17.2	0	171±25
	ROSC 1 h	102±4	7.36±0.03	39.6±3.5	134.7±14.9	254±11	173±21
	ROSC 3 h	108±3	7.33±0.03	38.2±3.1	142.7±17.1	277±12	169±23
CA	Baseline	107±8	7.45±0.06	40.5±3.4	145.4±18.2	256±11	176±23
	VF	22±2^a^	6.63±0.12^a^	90.7±7.8^a^	10.3±4.2^a^	0	520±18^a^
	ROSC 1 h	95±4	7.43±0.05	45.7±3.9	125.9±12.7	283±13	356±25^a^
	ROSC 3 h	98±5	7.40±0.08	40.8±5.0	145.7±7.3	271±14	289±21
CA+SE	Baseline	103±4	7.38±0.05	39.3±4.1	145.5±11.8	261±12	175±23
	VF	23±3^a^	6.83±0.13^a^	95.6±7.2^a^	9.7±2.3^a^	0	487±23^a^
	ROSC 1 h	98±9	7.32±0.05	39.6±3.7	125.6±7.9	267±15	347±17^a^
	ROSC 3 h	96±4	7.36±0.04	41.1±2.3	146.1±8.4	259±12	223±19
Sham	Baseline	109±6	7.38±0.03	39.5±4.3	147.7±15.6	257±12	165±19
	VF	111±3	7.41±0.02	42.7±2.7	158.1±18.3	0	171±21
	ROSC 1 h	106±4	7.39±0.03	41.4±4.1	140.7±13.2	277±17	174±21
	ROSC 3 h	112±3	7.37±0.03	39.6±4.1	143.5±13.5	263±12	182±22
CA	Baseline	113±8	7.40±0.06	43.5±3.4	147.4±16.8	252±11	183±25
	VF	21±2^a^	6.45±0.11^a^	92.7±6.7^a^	9.3±3.5^a^	0	556±38^a^
	ROSC 1 h	97±5	7.40±0.05	43.7±3.2	123.2±134	243±9	363±25^a^
	ROSC 3 h	93±2	7.38±0.08	42.3±3.4	147.7±8.8	261±10	232±21
CA+SE+SAL	Baseline	108±4	7.42±0.04	41.3±4.2	143.5±12.1	271±15	179±21
	VF	18±2^a^	6.63±0.14^a^	93.6±6.2^a^	10.7±4.3^a^	0	572±19^a^
	ROSC 1 h	95±4	7.38±0.04	38.6±3.2	135.6±6.2	263±12	341±15^a^
	ROSC 3 h	106±9	7.39±0.04	42.3±3.6	143.1±7.4	258±10	211±14
CA+Se+W	Baseline	114±5	7.43±0.03	41.6±4.9	151.2±5.5	262±11	172±21
	VF	20±2^a^	6.68±0.14^a^	94.4±7.2^a^	12.3±3.4^a^	0	578±35^a^
	ROSC 1 h	100±3	7.36±0.03	40.5±3.1	125.7±5.6	250±10	349±23^a^
	ROSC 3 h	102±4	7.41±0.02	38.8±5.2	143.9±6.7	245±9	234±17
CA+ W	Baseline	112±3	7.42±0.03	40.7±5.8	109±4	271±9	169±25
	VF	21±3^a^	24±3^a^	20±2^a^	19±3^a^	0	568±34^a^
	ROSC 1 h	107±5	97±4	95±3	97±2	262±10	357±27^a^
	ROSC 3 h	98±6	99±5	96±4	100±4	265±11	217±12

*All values are the mean ± SD. Arterial blood gas tensions include PaO_2_, PaCO_2_, pH, and Glc. MAP, mean arterial pressure; PaO_2_, arterial oxygen pressure; PaCO_2_, arterial carbon dioxide pressure; HR, heart rate; Glc, glucose*.

a *Controlled parameter*.

### Survival of rats

**Figure 2:**
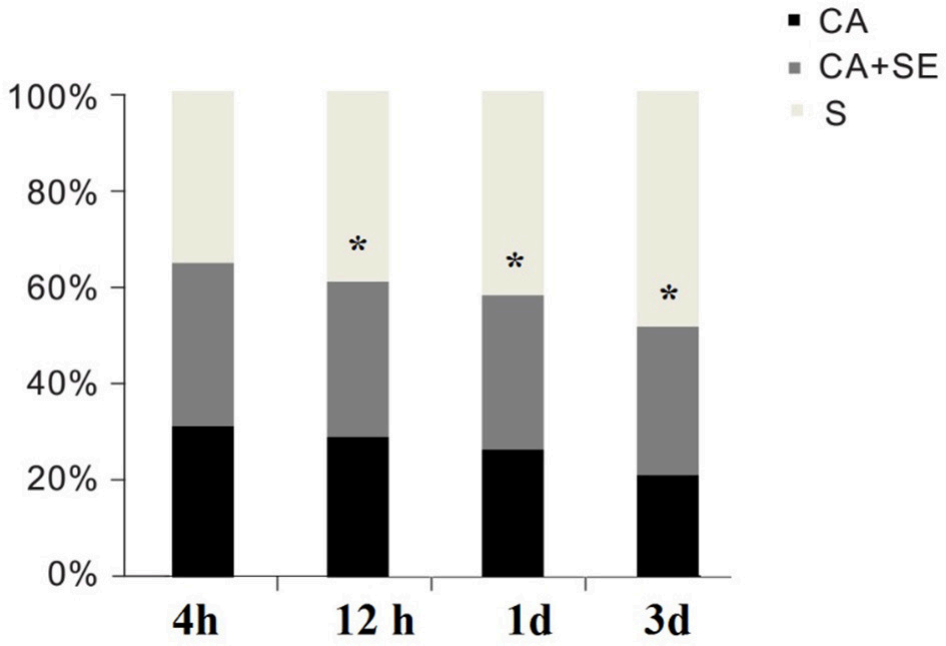
**Survival rates for the S, CA, and CA+SE groups**. ^*^P < 0.05 vs. CA group. S, sham group; CA, cardiac arrest group; SE, Sevoflurane.

Ventricular fibrillation was successfully induced in all rabbits in the CA group and the two CA+SE groups. There were no significant differences in the resuscitation parameters among the groups (*P* > 0.05; Table 3). The 72 h survival rates in the sham group, CA group, CA+SE 100% (6/6), 43.75% (7/16), 62.5%, and (9/16), respectively. The survival rate of rabbits in the sham group was significantly different from those in the 2 groups with CA. In addition, a significant difference in the survival rate was found between the CA+SE and the CA group (*P* < 0.05; Figure 2). The number of deaths in different groups at each time point has shown in Table 4.

**Table 3:** **Comparison of resuscitation parameters in the 2 groups witb CA**.

**Group**	**ICAT (s)**	**DOCPR (s)**	**DF**	**AF**	**MVT (min)**
CA	34.35 ± 13.16	212.64 ± 73.47	0.73 ± 0.49	4.84 ± 1.72	38.72 ± 11.08
CA+SE	37.58 ± 13.89	203.34 ± 64.85	0.75 ± 0.62	4.55 ± 1.59	33.53 ± 12.84

*ROSC, restoration of spontaneous circulation; ICAT, induce CA time (s); DOCRP, duration of CRP (s); DP, defibrillation frequency; AF, administration frequency, MVf, mechanical ventilation time (mm)*.

**Table 4:** **The number of deaths in different groups at each time point**.

**Group/Period**	**Number of deaths**				**Total number of deaths**
	**4 h**	**12 h**	**24 h**	**72 h**	
**EXPERIMENT 1**					
S	0	0	0	0	0
CA	2	3	3	2	10
CA+SE	0	2	2	2	6
**EXPERIMENT 2**					
S	0	0	0	0	0
CA	2	4	3	3	12
CA+SE+SAL	0	2	3	0	5
CA+SE+W	2	3	4	3	12
CA+W	1	3	4	2	10

### Sevoflurane treatment ameliorate neuronal injury in the hippocampus

**Figure 3:**
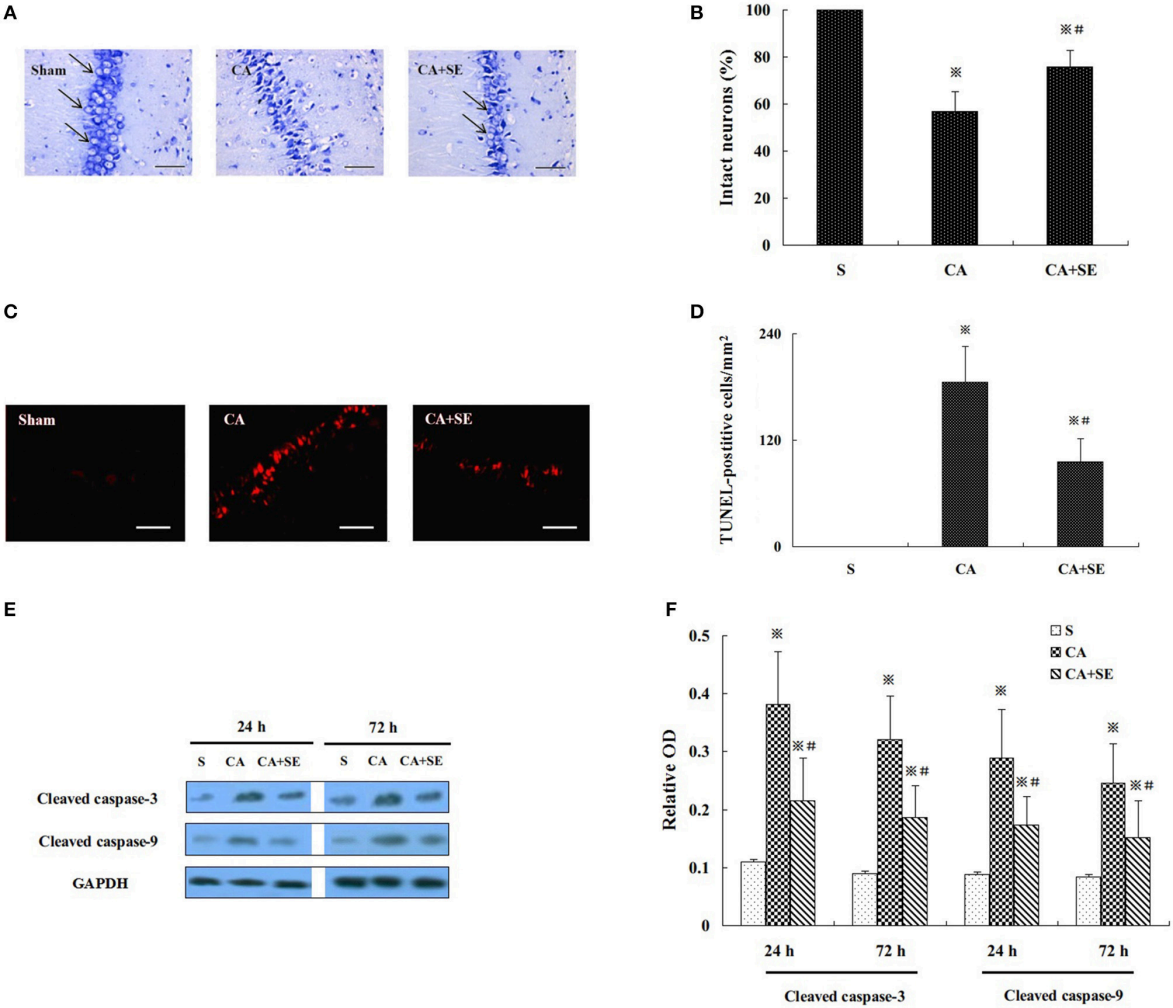
**Sevoflurane treament ameliorate cerebral ischemic injury in CA model. (A)** Nissl staining showed the pathological changes in CA1 region; Magnification 400×, Bar = 100 μm. **(B)** The percentage of intact neurons in CA1 region after 72 h reperfusion in different groups (*n* = 6). **(C)** TUNEL staining was performed on the CA1 region. Magnification is 400×. Scale bar = 100 μm. **(D)** The statistical analysis of apoptotic neurons in CA1 region of different group (*n* = 6). **(E)** Western blot analysis showed cleaved caspase-3 and -9 expression in the hippocampus of animals treated with or without sevoflurane. **(F)** Quantification of cleaved caspase-3 and -9 expression at 24 and 72 h after global cerebral ischemia. Data are expressed as mean ± *S.D*. *n* = 6–8 rats for each group. ^※^*P* < 0.05 vs. the sham group; ^#^*P* < 0.05 vs. the CA group.

Delayed neuronal death in the hippocampus was detected at 72 h after reperfusion. Compared with the sham group (100%), the number of neurons in other two groups with CA was significantly reduced (*P* < 0.01). In the CA group, the number of neurons was significantly reduced (46.98 ± 8.36%), and the nucleus was compressed; a Nissl body was not found in the cytoplasm. Compared with the CA group, many neurons with normal nucleus were found in the CA+SE group (70.62 ± 6.23%) and there were many Nissl bodies observed in the cytoplasm, suggesting that sevoflurane preserved more intact neurons (Figures 3A,B).

TUNEL staining showed many strongly TUNEL-positive apoptotic cells in the CA group, and a few weakly positive cells in the CA+SE groups. However, no TUNEL-positive cells were found in the sham group. Compared with the sham group, the TUNEL positive cells were significantly increased in the CA and CA+SE groups (*P* < 0.01). However, the TUNEL positive cells in the CA+SE group were significantly lower than those in the CA group (185.36 ± 40.63/mm^2^ in CA group vs. 95.69 ± 25.68/mm^2^ in CA+SE group, *P* < 0.05, Figures 3C,D).

Figure 3E represents the typical results of Western blot analysis of cleaved caspase-3 and -9 at 24 and 72 h after reperfusion, respectively. There was low expression of cleaved caspase-3 and -9 in the sham group. The quantitative analysis in Figure 3F indicated that there were higher cleaved caspase-3 and -9 expressions in the CA group than in the CA+SE group, suggesting that sevoflurane post-conditioning can efficiently attenuate neuronal apoptosis (*P* < 0.05).

### Sevoflurane post-conditioning promoted mitochondrial biogenesis after ischemic reperfusion

**Figure 4:**
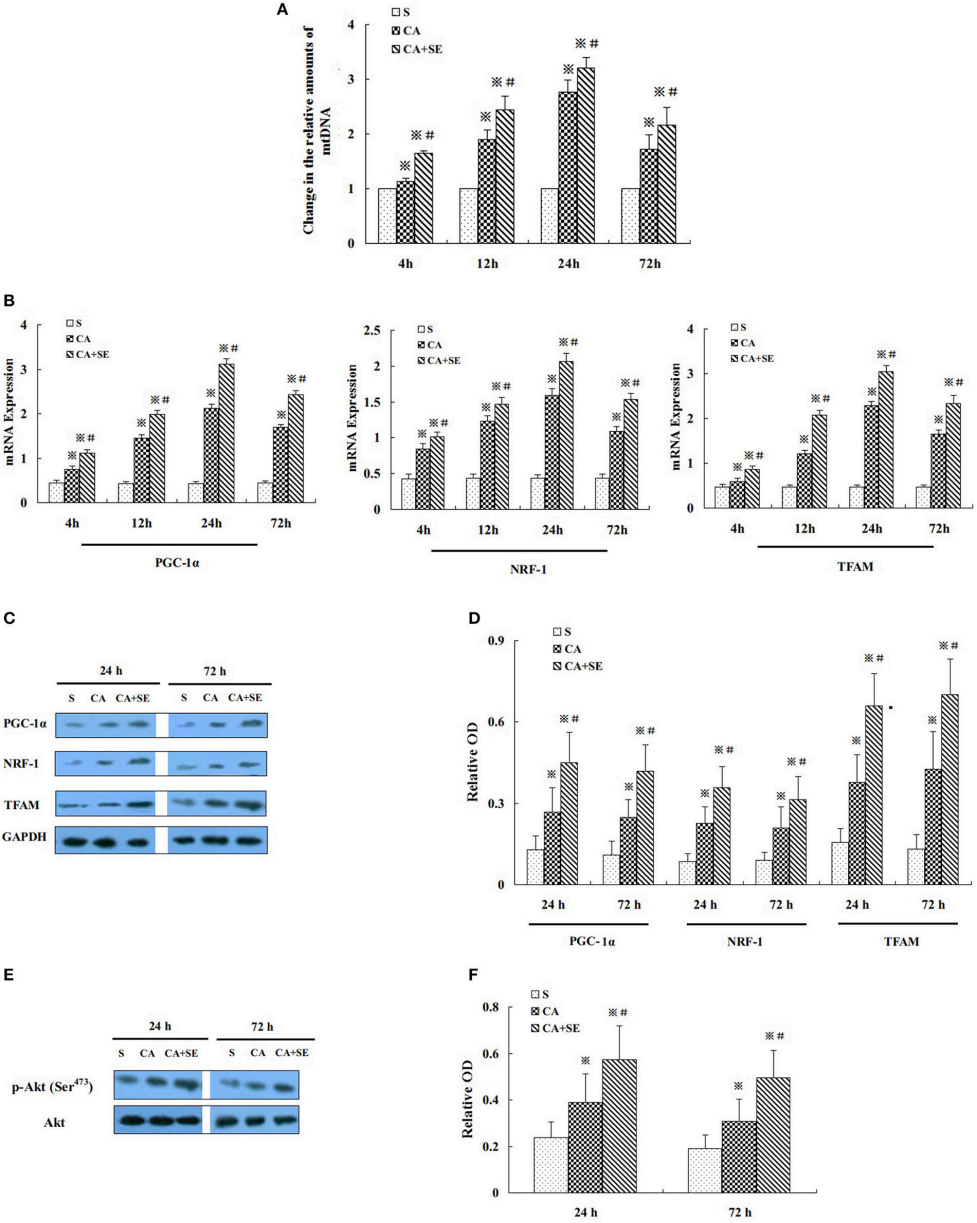
**Effect of sevoflurane post-conditioning on hippocampal neuronal mitochondrial biogenesis after global cerebral ischemic injury. (A)** The relative amounts of hippocampal mtDNA were measured using quantitative real-time PCR in three groups. **(B)** The histogram shows the quantitative measurement of real-time PCR products of mitochondrial biogenesis factors obtained using densitometric analysis. **(C)** Western blot images for PGC-1ɑ, NRF-1, and TFAM in rats in the presence and absence of sevoflurane; **(D)** Quantitative analysis of PGC-1ɑ, NRF-1, and TFAM expression levels. **(E)** Western blots analysis for p-Akt in the hippocampus of different group at 24 and 72 h after reperfusion. **(F)** Quantitative analysis for the level of p-Akt in thehippocampus. Data are expressed as mean ± *S.D*. *n* = 6–8 rats for each group. ^※^*P* < 0.05 vs. the sham group; ^#^*P* < 0.05 vs. the CA group.

Mitochondrial DNA (mtDNA) replication is necessary for mitochondrial biogenesis and is a typical marker for this process. Hence, we detected the relative abundance of mitochondria in the reperfused cerebral hippocampal tissue by measuring the mtDNA content using quantitative real-time PCR. The mtDNA content was examined 4, 12, 24, and 72 h after reperfusion. Compared with the sham group, the mtDNA content was increased in the CA and CA+SE groups in a time-dependent manner and peaked at 24 h (*P* < 0.05). In the CA+SE group, the mtDNA content were 1.4-, 1.5-, 1.3- and 1.2-fold higher, respectively, than those of the CA group at each time point (*P* < 0.05, Figure 4A).

Additionally, to determine the intrinsic specificity in mitochondrial biogenesis, we examined PGC-1α, NRF-1, and TFAM -transcription factors that have been reported to control mitochondrial gene expression. The mRNA expression levels of PGC-1α, NRF-1and TFAM were examined at 4 h, 12, 24, and 72 h after reperfusion by quantitative real-time PCR. Low levels of PGC-1α, NRF-1, and TFAM mRNA were observed in the hippocampus of the sham group. Compared with the sham group, PGC-1α, NRF-1, and TFAM mRNA were significantly induced in CA and CA+SE groups in a time-dependent manner and peaked at 24 h (*P* < 0.05). However, in contrast with the CA group, sevoflurane post-conditioning markedly increased the expression of PGC-1α, NRF-1, and TFAM mRNA in the hippocampus after 72 h of reperfusion (*P* < 0.05, Figure 4B).

To gain additional evidence for sevoflurane protective effect on the mitochondrial biogenesis, the protein expression levels of PGC-1α, NRF-1, and TFAM were investigated using Western blot analysis. Protein levels of PGC-1α, NRF-1, and TFAM were significantly increased at 24 and 72 h after reperfusion, when compared to the sham group (*P* < 0.05). Compared with the CA group, administration of sevoflurane significantly elevated the protein expression levels of PGC-1α, NRF-1, and TFAM (*P* < 0.05, Figures 4C,D).

Additionally, PI3K and its downstream protein Akt are known to play an important role in survival against ischemia/reperfusion-induced brain damage. To investigate whether Akt is involved in sevoflurane-induced neuroprotection, we evaluated the expression of Akt and its activated, phosphorylated form [p-Akt (Ser^473^)]. Total Akt expression was comparable in all experimental groups. p-Akt (Ser^473^) expression was significantly increased in the CA and CA+SE groups compared with sham-operated controls (*P* < 0.05). However, the expression of p-Akt (Ser^473^) was apparently increased by sevoflurane post-conditioning, when compared with the CA group (*P* < 0.05, Figures 4E,F).

### Effect of sevoflurane post-conditioning on mitochondrial antioxidant protein expression after reperfusion

**Figure 5:**
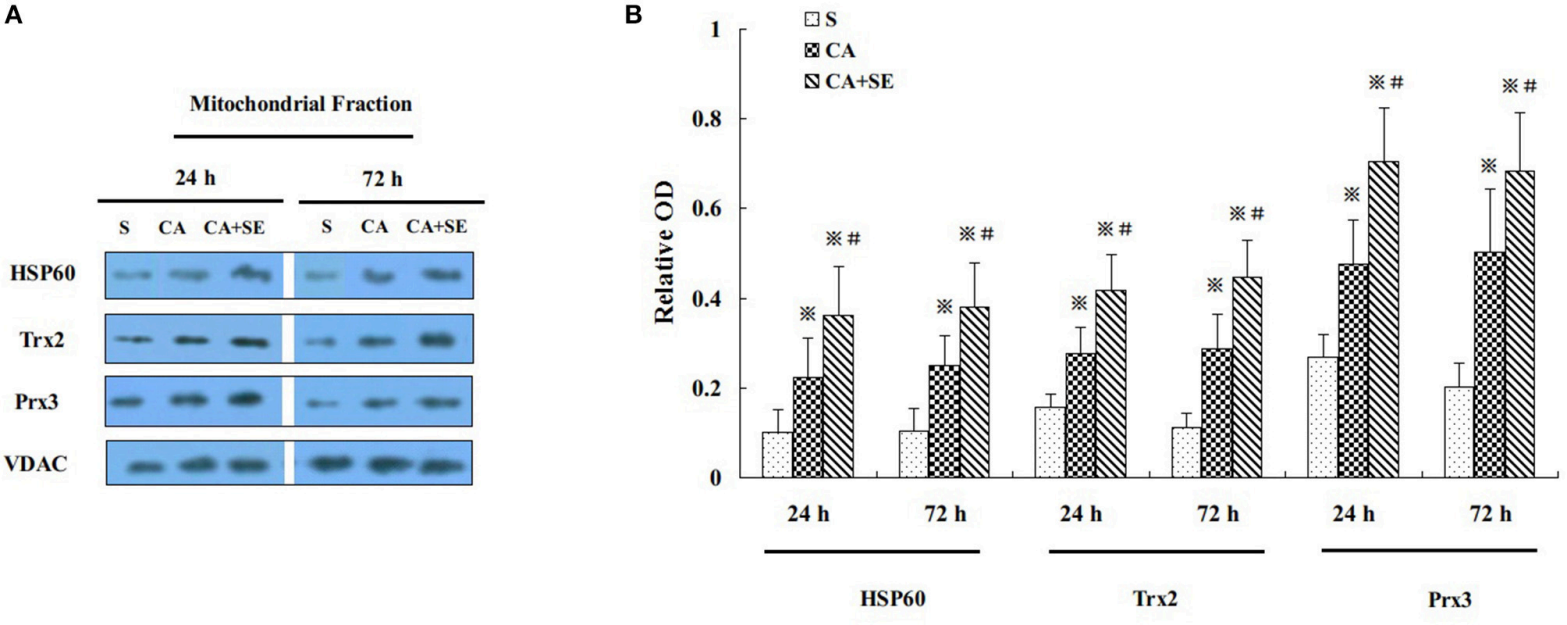
**Effect of sevoflurane post-conditioning on mitochondrial antioxidnt protein expression. (A)** Representative Western blots showing HSP60, Prx3, and Trx2 protein levels in rats in the presence and absence of sevoflurane; **(B)** Graph showing semiquantitative analysis showing the protein levels of HSP60, Prx3, and Trx2. Data are expressed as mean ± *S.D*. *n* = 6–8 rats for each group. ^※^*P* < 0.05 vs. the sham group; ^#^*P* < 0.05 vs. the CA group.

To gain additional evidence for the neuroprotection induced by sevoflurane post-conditioning, the expression level of several proteins normally enriched in mitochondria was investigated. Heat-shock protein 60 (HSP60), an abundant protein located primarily in mitochondria, was found to rise significantly 24 and 72 h after reperfusion in the CA+SE group, when compared with the CA group. Similarly, mitochondria-specific antioxidant enzymes such as peroxiredoxin 3 (Prx3) and thioredoxin 2 (Trx2) were examined by Western blot analysis at 24 and 72 h after reperfusion in the mitochondrial fraction. Compared with the CA group, the sevoflurane post-conditioning group had much higher protein expression of mitochondrial Prx3 and Trx2 at 24 and 72 h after reperfusion (Figures 5A,B).

### Mitochondrial ROS (mROS) formation and evaluation of mitochondrial integrity

**Figure 6:**
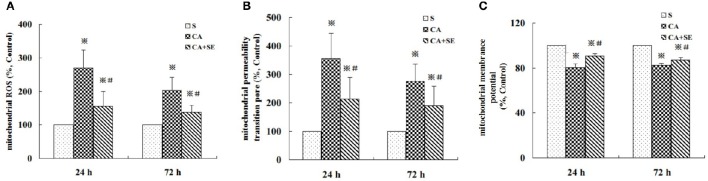
**Effect of sevoflurane post-conditioning on ROS generation, the degree of MPTP opening and MMP in the hippocampus at 24 and 72 h after reperfusion. (A)** mROS production was measured by using DCFH -DA at 24 and 72 h after reperfusion. **(B,C)** Mitochondrial integrity was measured in hippocampal cells following treatment with or without sevoflurane. The results are expressed as percentage of the sham group and presented as mean ± *S.D*. *n* = 6–8 rats for each group. ^※^*P* < 0.05 vs. the sham group; ^#^*P* < 0.05 vs. the CA group.

To determine if the protective effect of sevoflurane post-conditioning is due to a reduction in ROS production, mROS production was measured by using DCFH-DA at 24 and 72 h after reperfusion. As shown in Figure 6A, ROS production in mitochondria of hippocampal neurons in the CA group markedly increased to 269.7% at 1 day, then gradually decreased to 203.5% at 3 days compared to sham group. On the other hand, sevoflurane exhibited mROS production levels of 156.8% at 1 day and 137.3% at 3 days compared to sham group, which suggested that sevoflurane was able to inhibit the generation of mROS in hypoxic hippocampal neurons.

To determine mitochondrial integrity, we detected the opening of mitochondrial permeability transition pore (MPTP), as well as mitochondrial membrane potential (MMP). Opening of the MPTP is important for mitochondrial events leading to programmed cell death. As compared to the sham group, the activity of MPTP in hippocampal neurons significantly increased in the CA group (356.8% at 1 h and 276.1% at 3 days, *P* < 0.05) and sevoflurane post-conditioning could decline the activity of MPTP in hippocampal neurons (214.5% at 1 day and 189.2 at 3 days, *P* < 0.05, Figure 6B).

The loss of MMP is the result of the opening of permeability transition pores. As shown in Figure 6C, global ischemic injury induced a significant loss of MMP in the CA group in mitochondria hippocampal tissue (80.25% at 1 h and 82.37% at 3 days, *P* < 0.05). However, sevoflurane post-treatment rescued markedly the loss of MMP in the therapy group (90.63% at 1 h and 87.25% at 3 days, *P* < 0.05).

### Sevoflurane-delayed post-conditioning was mediated by the PI3K/Akt survival pathway

**Figure 7:**
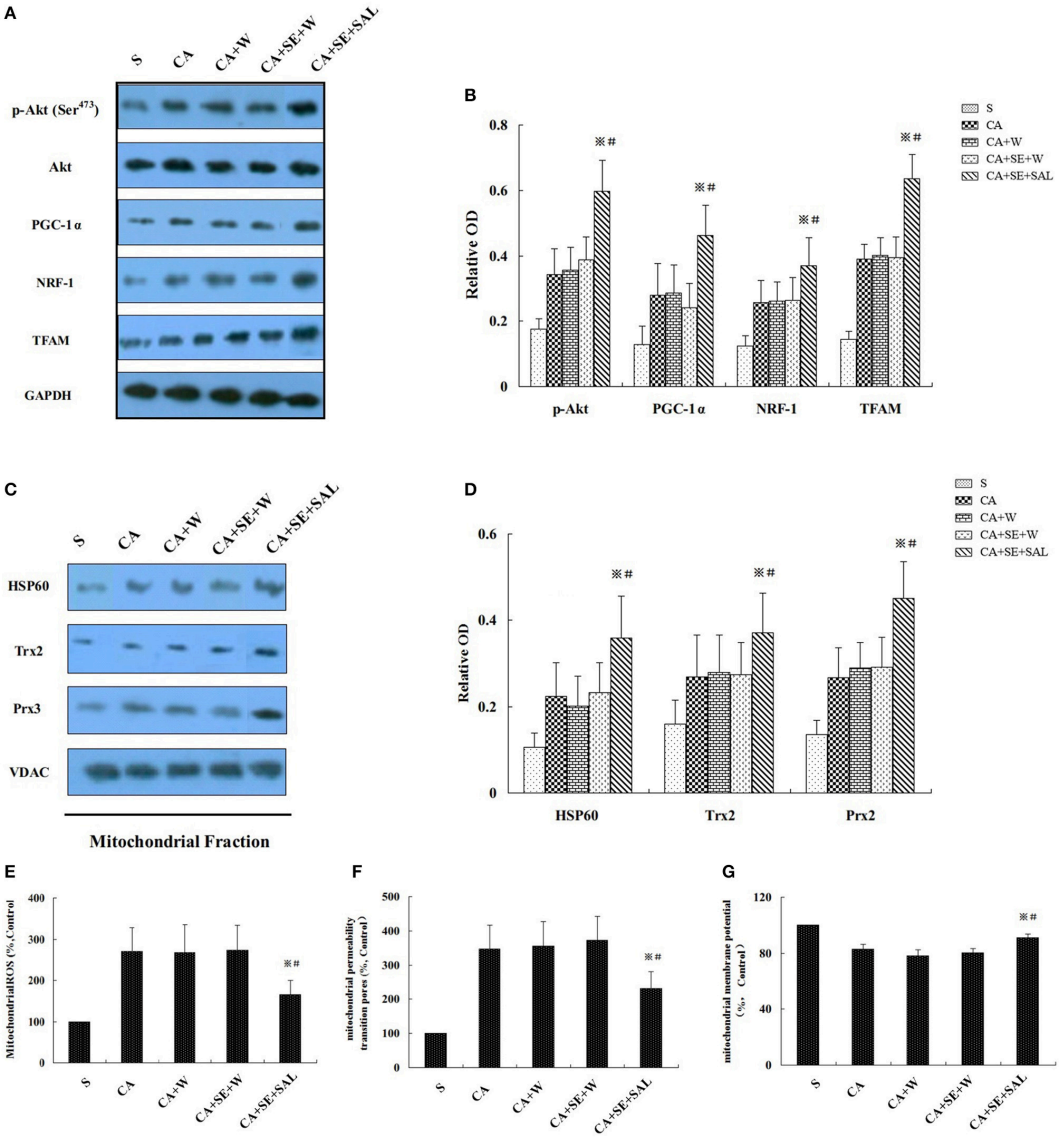
**Sevoflurane enhanced neuron resistance to global cerebral injury by the PI3K/AKT signaling pathway. (A,C)** Western blots analysis for p-Akt, mitochondrial biogenesis related proteins, and mitochondria-specific antioxidant enzymes at 24 h after reperfusion. **(B,D)** Quantitative analysis for the level of p-Akt, mitochondrial biogenesis-related proteins and mitochondria-specific antioxidant enzymes. **(E–G)** Effect of wortmannin on sevoflurane post-conditioning-inhibited mROS generation, loss of MMP and opening of MPTP. Data are expressed as mean ± *S.D*. *n* = 6–8 rats for each group. ^※^*P* < 0.05 vs. SE+W group; ^#^*P* < 0.05 vs. the CA group.

Similarly as aforementioned, sevoflurane significantly increased p-Akt (Ser^473^), mitochondrial biogenesis-related proteins such as PGC-1α, NRF-1 and TFAM, and mitochondria-specific antioxidants including HSP60, Trx2, and Prx2 in hippocampal tissue, compared with the sham and control groups at 24 h after reperfusion. Administration of PI3K inhibitor wortmannin decreased the elevation of p-Akt level, mitochondrial biogenesis-related proteins and mitochondria-specific antioxidants induced by sevoflurane post-conditioning (*P* < 0.05, Figures 7A–D). However, total Akt expression was not obviously changed in all groups (*P* > 0.05). There was no significant difference in protein levels between the control group and the wortmannin alone (CA+W) group (*P* > 0.05).

Sevoflurane post-conditioning also ameliorated mitochondrial ROS generation and restored the mitochondrial integrity. Nevertheless, wortmannin treatment eliminated the beneficial biochemical processes of sevoflurane, shown as increased mitochondrial ROS generation, and loss of MMP and opening of MPTP at 24 h of reperfusion (*P* < 0.05, Figures 7E–G). There was no significant difference in all the parameters between the control group and the CA+W group (*P* > 0.05).

## Discussion

Although a continually improved outcome after global brain ischemia induced by CA or cardiopulmonary bypass surgery has been achieved in patients, there has been no revolutionary breakthrough. Therefore, understanding the mechanisms responsible for brain injury due to global ischemia and finding new neuroprotective strategies are critical and will be useful in clinical settings (Yannopoulos et al., 2011).

Growing evidence has shown that treatment with sevoflurane, a volatile anesthetic agent, before or after ischemia can improve neuronal recovery after the insult (Ye et al., 2012a,b,c, 2015). Hence, sevoflurane may be ideal chemical preconditioning or post-conditioning agents since they are safe and broadly used in clinical practice. Until now, the pro-apoptotic pathway has been the main pathogenesis in neuronal cell death induced by cerebral ischemic insult. In the current paper, we demonstrated that sevoflurane post-conditioning efficiently increased post-ischemic survival rates. Furthermore, sevoflurane post-conditioning significantly attenuated the activation of caspase-3 and -9 to reduce post-ischemic neuronal apoptosis. Therefore, our data suggest that sevoflurane post-conditioning is remarkably effective in preventing neuronal apoptosis in the hippocampus in a model of CA-induced global cerebral ischemia.

Accumulating evidence has strongly implicated that mitochondria may be involved in causing ischemia-related cell death (Doll et al., 2015; Lu et al., 2015; Jin et al., 2016). Normal mitochondrial function plays an essential role in promoting neuronal survival after ischemic reperfusion injury. Previous studies have found that improved mitochondrial biogenesis can reduce neuronal damage during cerebral ischemia through stimulating the renewal of mitochondrial function (Liu et al., 2014; Pan et al., 2014). Although sevoflurane post-conditioning has the ability to prevent cerebral ischemia-induced apoptosis, its effect on mitochondrial biogenesis and function has not been well-studied. Mitochondrial DNA (mtDNA) replication is necessary for mitochondrial biogenesis and is a typical marker for this process. Moreover, mtDNA quantity is an indicator of mitochondria content. We measured the amount of mtDNA using real-time PCR to dissect the effect of sevoflurane on mitochondrial biogenesis. We showed that in contrast to ischemic stimulation, sevoflurane post-conditioning significantly increased the amount of mtDNA. Additionally, mitochondrial biogenesis is a highly regulated process that requires the participation of both the nuclear and mitochondrial genomes. To investigate the molecular mechanisms that might be responsible for regulating mitochondrial biogenesis after sevoflurane post-conditioning, we examined three transcription factors considered essential for mitochondrial gene expression. PGC-1α is a co-transcriptional regulation factor that induces mitochondrial biogenesis by activating different transcription factors, including nuclear respiratory factors 1 and 2 proteins (NRF-1 and NRF-2; Kleiner et al., 2009; Sharma et al., 2015). Furthermore, PCG-1α indirectly induces mtDNA replication and transcription by increasing the expression of mtDNA regulatory genes, including mitochondrial transcriptional factor A (TFAM; Wu et al., 1999; Kleiner et al., 2009). It is known that the transcriptional activity of NRF-1 is enhanced by PCG-1α in mitochondrial biogenesis (Escrivá et al., 1999; Scarpulla, 2002; Ping et al., 2015), and the expression of TFAM is, at least partially, under the control of NRF-1 (Escrivá et al., 1999; Biala et al., 2010). Up-regulation of PCG-1α coordinates gene activation and facilitates mitochondrial biogenesis. Numerous studies have demonstrated that ischemic or hypoxic injury increases mitochondrial biogenesis (Pan et al., 2014; Wang et al., 2014; Xie et al., 2014). Thus, to further support sevoflurane's protective effect on mitochondrial biogenesis, the gene and protein levels of three critical genes for the transcriptional regulation of mitochondrial biogenesis, PGC-1α, NRF-1, and TFAM, were investigated. Compared with the global cerebral ischemia group, the sevoflurane post-conditioning group had much higher gene and protein expression levels of PGC-1α, NRF-1, and TFAM in the hippocampus after reperfusion. Therefore, in this current study, comparable to the increase in mtDNA, an increase in the expression of three mitochondrial biogenesis-related proteins was found. Actually, mitochondrial biogenesis can be induced by ischemic or hypoxic damage, and this enhanced mitochondrial biogenesis may increase the abundance of mtDNAs and maintain their aerobic set-point in case of declining function. In brief, our findings suggest that sevoflurane can effectively improve mitochondrial biogenesis through facilitating mtDNA amplification and elevating PGC-1α, NRF-1, and TFAM gene and protein expression.

Recently, substantial evidence has indicated that the Trx and Prx redox system is associated with neuronal damage and neuroprotective effects via the regulation of oxidative stress in brain ischemia (Hwang et al., 2010; Lee et al., 2015). To gain additional evidence for sevoflurane post-conditioning-induced neuroprotection against cerebral ischemia, the expression level of several proteins normally enriched in mitochondria was investigated. Peroxiredoxin 3 (Prx3) is exclusively found in mitochondria and plays a role in the antioxidant defense system and homeostasis within mitochondria (Hwang et al., 2010; Du et al., 2014). Thioredoxin 2 (Trx2) is expressed with a mitochondrial leading sequence and transported specifically into mitochondria, playing an essential role in cell survival and mitochondria-mediated apoptosis (Ma et al., 2013; Lee et al., 2015). Among the subtypes of Trx and Prx, Prx3 and Trx2 are exclusively expressed in the mitochondrial compartment, and are involved in the control of the antioxidant defense system, cell survival, and apoptosis. A recent paper pointed out that treatment with Prx3 and Trx2 in ischemic brains showed a potent neuroprotective effect against ischemic damage by reducing lipid peroxidation and mitochondria-mediated apoptosis (Hwang et al., 2010). Additionally, the mitochondrial chaperonins composed of heat shock protein 60 (HSP60) are the major sites of protein folding in mitochondria. HSP60 is an abundant protein located primarily in mitochondria, with only 15–20% normally found in the cytosol (Sun et al., 2014). In addition, the upregulation of HSP60 is indicative of mitochondrial biogenesis (Li et al., 2014). Our findings showed that Prx3, Trx2, and HSP60 in the mitochondria of the sevoflurane group was much higher than those in the CA group, suggesting that amelioration of cerebral ischemic injury by sevoflurane may be attributed to its up-regulation of mitochondrial antioxidases.

Although we have shown that sevoflurane post-conditioning triggered significant protection against CA-induced global cerebral ischemia by preserving mitochondrial biogenesis and promoting mitochondrial antioxidant enzymes, the underlying mechanism for sevoflurane's neuroprotection effect remains to be elucidated. On the other hand, mitochondria are also regarded as the critical regulators of cell death and major cellular sources of ROS, which causes damage to mtDNA in neuronal cells (Watabe et al., 1997; Tanaka et al., 2002). Surplus amount of ROS generation may result in the loss of mitochondrial membranes potential (MMP) and the opening of mitochondrial permeability transition pore (MPTP) and cause mitochondrial dysfunction, which in turn modulates pro-apoptotic protein activation (Gupta and Knowlton, 2002; Yin et al., 2008). Therefore, we determined the effects of sevoflurane post-conditioning on mROS production, MMP and the opening of MPTP. Sevoflurane caused a decrease in mROS generation, MPTP opening, as well as in salvage of MMP indicating that sevoflurane could retain mitochondrial integrity post-ischemia. In fact, the Trx2 and Prx3 redox system and HSP60 are important in mitochondrial function by reducing oxidative stress via regulating mROS levels.

Consistent with other studies, we have also demonstrated that activation of the PI3K/Akt pathway mediated salvage of neurons against infarction during post-conditioning by the volatile anesthetic agent sevoflurane. It is interesting to note that wortmannin, a PI3K inhibitor, abolished the neuroprotective effect of sevoflurane post-conditioning by significantly decreasing the protein levels of p-Akt (Ser^473^), mitochondrial biogenesis-related proteins (PGC-1α, NRF-1, and TFAM), and mitochondria-specific antioxidant enzymes, as well as increasing mROS production and aggravating mitochondrial integrity. These results suggested that sevoflurane might elicit the protective role in global cerebral ischemic injury by regulating the PI3K/Akt survival pathway. Actually, PI3K/Akt signaling was found to be reversely associated with mitochondria oxidative stress and activation of PI3K/AKT signaling reduced ROS production (Dai et al., 2013; Zhu et al., 2015). Furthermore, phosphorylated Akt (p-Akt) not only acts to inhibit pro-apoptotic factors and stimulate anti-apoptotic factors, but also inactivates mitochondria-based oxidative stress (Wang et al., 2015; Xu et al., 2015). Hence, our findings support the hypothesis that sevoflurane ameliorated mROS formation post-ischemia by up-regulation of Trx2, Prx3, and HSP60 expression and interrupted the subsequent cascade reaction, which eventually resulted in improving mitochondrial biogenesis and integrity, possibly via the activation of the PI3K/Akt survival pathway.

Nevertheless, we have only shown that the PI3K/Akt survival pathway mediated acute (24 h) post-conditioning neuroprotection induced by sevoflurane. The long-term effect (up to 28 days post cerebral ischemia) of sevoflurane post-conditioning is still unclear, which may be a major limitation of this present study.

In conclusion, administration of sevoflurane after reperfusion was able to significantly induce the gene and protein levels of mitochondrial pro-survival proteins, including PGC-1α, NRF-1, and TFAM, up-regulate mitochondria-specific antioxidant enzyme expression, and restore mitochondrial integrity. In parallel, the level of cleaved caspase-3 and -9, two proteins involved in apoptotic cell death, and the number of damaged neurons in hippocampus sections were reduced in the presence of sevoflurane. Furthermore, the protective role of sevoflurane post-conditioning-triggered brain protection was mediated through PI3K/AKT signaling. Accordingly, this research may support a promising therapeutic avenue for the treatment of global cerebral ischemic injury during the preoperative period. Future studies will further investigate the *in vitro* mechanisms of sevoflurane post-conditioning.

## Author contributions

Conceived and designed the experiments: ZW, ZY, and QG. Performed the experiments: ZW, ZY, and GH. Analyzed the data: NW and EW. Contributed reagents/materials/analysis tools: NW, QG, and EW. Wrote the manuscript: ZY.

### Conflict of interest statement

The authors declare that the research was conducted in the absence of any commercial or financial relationships that could be construed as a potential conflict of interest.
